# Cytotoxicity comparison of Bio C Sealer against multiple root canal sealers

**DOI:** 10.4317/jced.59868

**Published:** 2023-02-01

**Authors:** Alba Tolosa-Monfà, Alma Veroni, Juan Blasi-Cabús, Maria-Lluisa Ballester-Palacios, Esther Berástegui-Jimeno

**Affiliations:** 1 Department of Endodontics, School of Dentistry, University of Barcelona, Barcelona, Spain; 2 Department of Pathology and Experimental Therapeutics, Faculty of Medicine and Health Sciences, University of Barcelona, Barcelona, Spain

## Abstract

**Background:**

The aim of this study was to compare the cytotoxicity of calcium silicate-based endodontic sealer, Bio-C® Sealer, with other calcium silicate-based sealers: BioRoot™ RCS, one silicon-based sealer combined with calcium silicate particles: GuttaFlow® Bioseal, one resin MTA-based root canal sealer: MTA Fillapex®, and an epoxy resin-based sealer: AH Plus®.

**Material and Methods:**

NIH 3T3 fibroblasts were cultured and sealers extracts were obtained. Cytotoxicity was evaluated by the MTS assay and the optical densities of the solutions were measured with a microplate reader. This study was designed with one sample for each control group and n=10 for each treatment group of the different sealants. The results were classified according to the degree of cell viability and underwent statistical analysis with the ANOVA test (*p*<0.05). The samples were examined under an inverted microscope to evaluate the effect of each sealer on fibroblast cell morphology.

**Results:**

Cells incubated with GuttaFlow® Bioseal extract showed the highest cell viability without statistically significant differences with the control group. BioRoot™ RCS and Bio-C® Sealer showed moderate (tending to slight) cytotoxicity and both AH Plus® and MTA Fillapex® showed severe cytotoxicity in comparison with the control group (*p*<0.05). AH Plus® and MTA Fillapex® were not significantly different from one another; nor was BioRoot™ RCS from Bio-C® Sealer. Microscope examination found that fibroblasts in contact with GuttaFlow® Bioseal and Bio-C® Sealer presented the most similar aspects to the control group both in terms of number and shape.

**Conclusions:**

Bio-C® Sealer showed moderate (tending to slight) cytotoxicity compared with the control group, GuttaFlow® Bioseal showed no cytotoxicity, BioRoot™ RCS moderate-slight cytotoxicity and AH Plus® and MTA Fillapex® severe cytotoxicity.

** Key words:**Biocompatibility, calcium silicate-based endodontic sealers, cytotoxicity, endodontic sealer.

## Introduction

During obturation of the canal system, unintentional extrusion of sealer through the apical constriction produces direct contact between the sealer and extracellular fluids and periapical tissues. This can induce inflammatory reactions and tissue damage depending on the degree of cytocompatibility of the sealer used (1). It can also produce adverse effects on repair mechanisms leading to subsequent clinical failure (2). For these reasons, it is important to choose materials with adequate physicochemical and biological properties, biocompatibility being one of the main characteristics for consideration.

Over the years, different types of sealers have been employed in endodontic treatment based on zinc oxide eugenol, calcium hydroxide, polydimethylsiloxane, silicon, epoxy resin, methacrylate resin, and more recently, calcium silicate-based sealers (3). Calcium silicate-based materials were introduced as root repair cements and root canal sealers and their popularity has increased in recent years due to their physicochemical and biological properties. The main advantages of calcium silicate-based sealers are their excellent biocompatibility and bioactive potential including a osteogenic capacity, which provokes a regenerative response (4) through their ability to form apatite thanks to the release of calcium hydroxide ions (5). These materials are composed of aluminum, zirconia particles, bioactive glass, calcium silicate, hydroxyapatite, and absorbable calcium phosphate, among others (6).

AH Plus® (Dentsply, York, PA, USA) is an epoxy resin-based root canal sealer, considered the gold standard due to its physical properties and high bond strength to dentin. Nevertheless, this sealer does not present bioactive properties (6).

MTA Fillapex® (Angelus, Santa Izabel, Londrina - Estado de Paraná, Brazil) is composed of salicylate resin, diluting resin, natural resin, bismuth oxide, silica nanoparticles, and calcium silicate combined with MTA. Although MTA enjoys excellent biocompatibility and bioactive potential, it has shown irritant effects on subcutaneous connective and bone tissue due to the presence of toxic components such as salicylate resin, diluting resin and silica (7). 

BioRoot™ RCS (Septodont, Saint-Maur-des-Fosses, France) is a calcium silicate-based sealer with antimicrobial properties due to calcium hydroxide release, which according to published research shows good results in terms of biocompatibility and bioactivity (4).

GuttaFlow® Bioseal (Coltene, Altstatten, Switzerland) is a silicon-based sealer with gutta-percha powder combined with calcium silicate particles (8). It has exhibited better biocompatibility in comparison with AH Plus® and MTA Fillapex®, as well as a bioactive capacity acting on periodontal ligament cells (6).

Bio-C® Sealer (Angelus, Londrina, PR, Brazil) is a premixed calcium silicate–based sealer composed of tricalcium silicate, dicalcium silicate, tricalcium aluminate, calcium oxide, zirconia oxide, silicon oxide, polyethylene glycol, and iron oxide (9). In recent studies, the sealer obtained a short setting time, alkalinization capacity, adequate flow and radiopacity, low volumetric change but higher solubility than the rates required by ISO standard 6876 (10) and good biocompatibility allowing rapid regression of the inflammatory reaction (9).

The aim of this study was to compare the cytotoxicity of new calcium silicate-based endodontic sealer, Bio-C® Sealer, with other calcium silicate-based sealers: BioRoot™ RCS, one silicon-based sealer combined with calcium silicate particles: GuttaFlow® Bioseal, one resin MTA-based root canal sealer: MTA Fillapex®, and an epoxy resin-based sealer: AH Plus®. The null hypothesis proposed was that there would not be significant differences in cytotoxicity between the different sealers.

## Material and Methods

This study investigated five sealers, two calcium silicate-based sealers (Bio-C® Sealer, BioRoot™ RCS), one silicon-based sealer combined with calcium silicate particles (GuttaFlow® Bioseal), one resin MTA-based root canal sealer (MTA Fillapex®), and one epoxy resin-based sealer (AH Plus®). This study was designed with one sample for each control group and n=10 for each treatment group of the different sealants.

-Cell culture 

First of all, NIH 3T3 fibroblasts were cultured in Dulbecco’s modified eagle medium (DMEM) complemented with 10% fetal bovine serum inactivated by heat (FBS) and 1% penicillin streptomycin (Pen-Strep). Cells were incubated at 37º and 95% humidity in a 5% CO2 atmosphere renewing the medium every 48 hours. To prepare them, a 10 cm culture plate with 80% confluence (% of the plate surface occupied by cells) was used, the culture medium (DMEM) was aspirated and the plate was washed several times with phosphate buffered saline (PBS). Then, 1 ml trypsin was applied to the plate to detach the cells, 20 ml of DMEM was again added, and the cells counted and placed in the wells in a 96-well plate, one to obtain 100% confluence and another 50%. These were incubated at 37º and 95% humidity in a 5% CO2 atmosphere for 24 hours.

-Endodontic sealer extracts and exposure to cells 

To obtain sealers extracts, they were prepared according to the manufacturers’ instructions, placing 0.5 ml of each sealer in a well in a 12-well sterile plate letting the sealer flow over the entire surface. These were placed in the incubator at 37º for 24 hours to allow all the materials to set and afterwards the specimens were exposed to UV rays for 30 minutes on each face in order to sterilize them. After this period, 5ml DMEM + 10% FCS + 1% Pen-Strep were placed in each well and incubated at 37º and 95% humidity in a 5% CO2 atmosphere for 24 hours to obtain the sealer extracts. At the end of the 24 hours, the extracts were filtered with a 0.2µm filter (Acrodisc® Syringe Filter 0.2µm Supor® Membrane Low Protein Binding Non-Pyrogenic) and 100µl of each were placed in the wells in contact with cells. Lastly, the samples were left to incubate at 37º and 95% humidity for 24 hours. For each sealer, 10 wells containing cells in contact with the sealer extracts were used and 3 containing Triton X-100 (TX) to determine the zero level or background. As a control group, ten wells were prepared with cells in DMEM without contact with any sealer extract.

After 24 hours exposure time, the effects of the sealers on fibroblast cell morphology was analyzed under an inverted microscope (Leica DMIRB, Wetzlar, Germany).

-Cytotoxicity assay 

Cytotoxicity was evaluated using a reactive that makes it possible to take a reading of metabolically active cells through a colorimetric reaction. The MTS assay (3-(4,5-dimethylthiazol-2-yl)-5-(3-carboxymethoxyphenyl)-2-(4-sulfophenyl)-2H-tetrazolium) is a tetrazolium compound that can be reduced by viable cells to generate formazan products that are soluble in cell culture medium. This conversion is accomplished by NADPH (nicotinamide adenine dinucleotide phosphate hydrogen) or NADH (nicotinamide adenine dinucleotide) produced by dehydrogenase enzymes in metabolically active cells. The quantity of formazan product is then measured by absorbance at 490nm and is directly proportional to the number of living cells in the culture. The higher the quantity of formazan, the greater the color saturation will be (optical density) and so the higher the number of metabolically active cells (11). So, 20µl of the reactive is placed in each well and cell reactions are evaluated every 15 minutes until 60 minutes, measuring optical densities with a microplate reader (Asys UVM 340, Biochrom) at a wavelength of 490nm.

The absorption value obtained with the control was considered as indicating 100% cell viability. Cytotoxicity was rated on the basis of cell viability relative to controls as: non-cytotoxic (>90% cell viability), slightly cytotoxic (60-90% cell viability), moderately cytotoxic (30-59% cell viability), and severely cytotoxic (<30% cell viability) (1,12).

-Statistical design and analysis 

This study was designed with a sample size of n=10 (due to its experimental nature) and a sample as control for each cement. Descriptive statistics for each variable were calculated: mean, standard deviation (s.d.), and quartiles q1, q2, q3 and q4. The sealers were compared using variance analysis, making estimations with a 95% confidence interval (CI); an alpha risk of 5% was set for hypothesis contrasts. To evaluate differences between the sealers, normal distribution of the variables was assumed testing homogeneity with the Levene test (*p*=0.0812). The subsequent comparison of the six groups via ANOVA or variance analysis, revealed that there are very significant differences between them, *p*<2e-16. Further analysis using the Tukey test allowed us to compare the pairwise differences between sealers and with the control group.

These analyses were performed using the statistical software IBM SPSS Statistics v.25 (SPSS Chicago,IL,United States), R Statisctical Software version 3.4.3, (R Foundation for Statistical Computing, Vienna, Austria)

## Results

**Figure 1:**
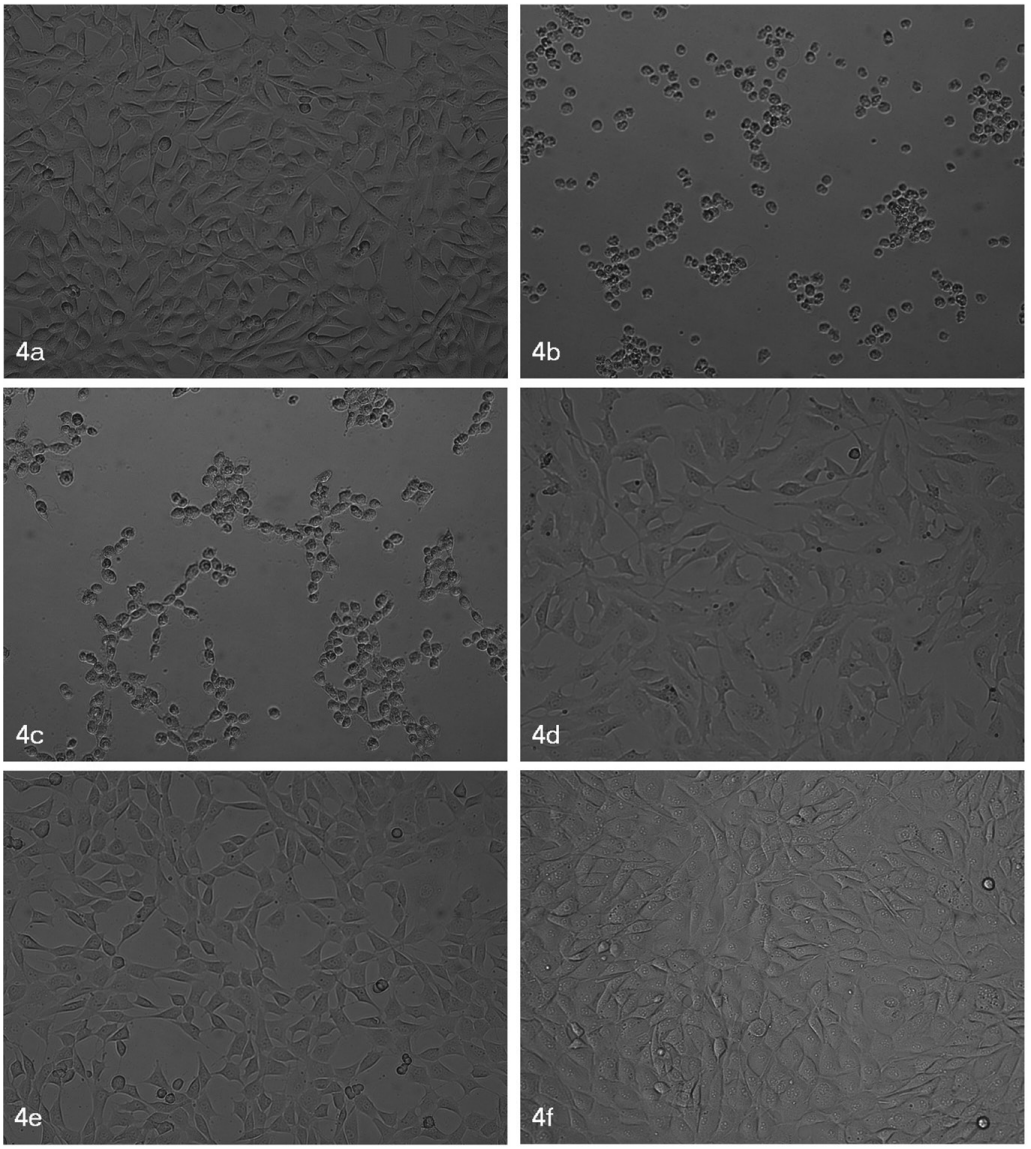
Morphological changes to fibroblasts after 24 exposure time to sealers. 4a: Control samples show normal fibroblast appearance; 4b: AH Plus® caused reduction in size and rounded shape; 4c: MTA-Fillapex® caused reduction in size, rounded shape, and chain formation; 4d: BioRoot™ RCS produced elongated prolongations; 4e: GuttaFlow® Bioseal showed similar appearance to control fibroblasts; 4f: Bio-C® Sealer produced similar appearance to control fibroblasts.

To evaluate the effects of sealers on fibroblast cell morphology, the samples were examined under an inverted microscope at 10X magnification after 24 hours exposure time (Fig. 1). It was found that fibroblasts in contact with GuttaFlow® Bioseal and Bio-C® Sealer presented an appearance more similar to the control group fibroblasts both in terms of numbers and shape, while cells in contact with AH Plus® and MTA Fillapex® presented a drastic reduction in size, a rounded shape, and a tendency to form chains in the case of MTA Fillapex®. For cells exposed to BioRoot™ RCS, fibroblasts prolongations appeared elongated. These morphological changes suggest cell suffering as a result of exposure to these sealers (13). In the case of AH Plus® and MTA Fillapex®, they underwent cell death indicated by the reduction in size and rounded shape, a finding that correlated to the cytotoxicity evaluations obtained.

When the MTS reactive was applied, different results were obtained according to the time of plate reading (15, 30, 45 and 60 minutes after reactive application) and cell confluence (100% or 50%). It was decided to regard the plates with 100% confluence at 60 minutes as providing the most reliable results. This time was chosen as the measurement presented optical densities (absorbance) that were sufficiently high but not yet saturated. The mean value was calculated for each sealer, deducting the result for the background obtained by applying Triton X-100 (TX).

Table 1 shows descriptive analysis of cell viability: mean values, standard deviation and percentiles.

**Table 1:**
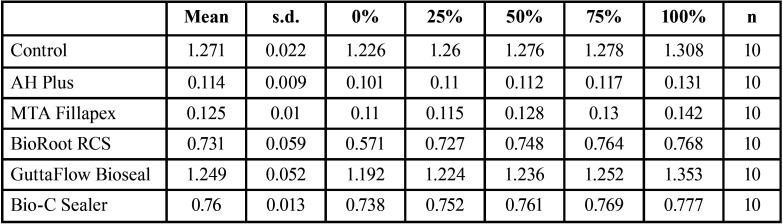
Descriptive analysis of cell viability: mean values, standard deviation (s.d.), percentiles, and sample size (n).

**Figure 2:**
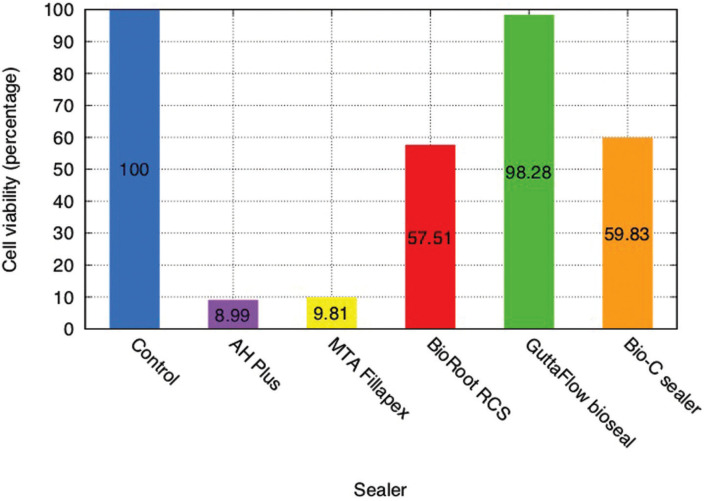
Results of cell viability in presence of sealer extracts 60 minutes after MTS test. Values are expressed as percentages in relation to control group.

The mean value for each sealer was converted into a percentage of living cells in relation to the control samples (Fig. 2).

Table 2 shows that all of them display statistically significant differences in comparison with the control group, the only exception is GuttaFlow®️ Bioseal.

**Table 2:**
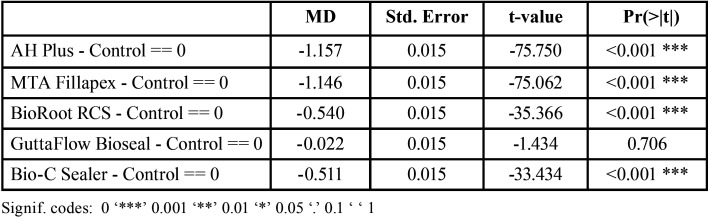
Paired comparisons between sealers and control group: differences in mean values (MD), standard error (Std.Error), t-value, and Pr(>|t|). The Pr(>|t|) column represents the p-value associated with the value in the t value column.

**Figure 3:**
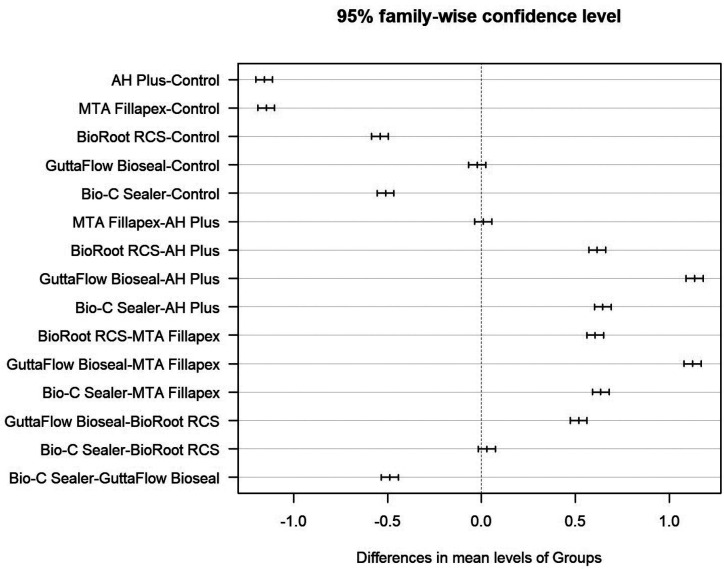
Paired comparisons between sealers and control: mean differences with 95% CI.

Table 3 shows paired comparisons between the sealers. Statistically significant differences were found between all pairs of sealers except AH Plus® and MTA Fillapex®; and BioRoot™ RCS and Bio-C® Sealer (Table 2, Fig. 3).

**Table 3:**
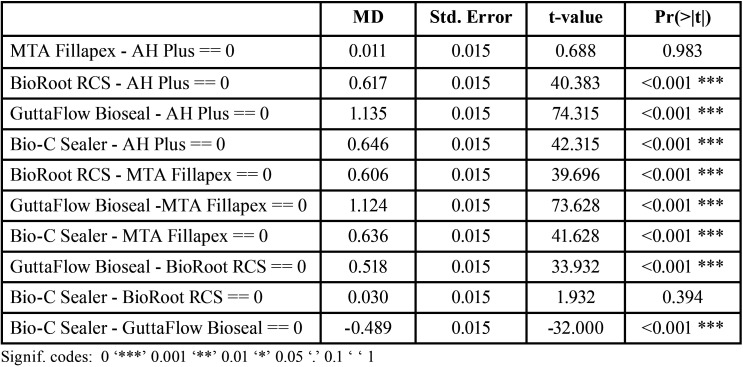
Paired comparisons between sealers: differences in mean values (MD), standard error (Std.Error), t-value, and Pr(>|t|). The Pr(>|t|) column represents the *p*-value associated with the value in the t value column.

These results indicate that cells incubated with extacts of GuttaFlow® Bioseal showed greater cell viability without significant differences in comparison with the control group (non-cytotoxic). BioRoot™ RCS and Bio-C® Sealer presented moderate (tending to slight) cytotoxicity, while both AH Plus® and MTA Fillapex® presented severe cytotoxicity in comparison with the control group. All the sealers except GuttaFlow® Bioseal, showed statistically significant differences in cytotoxicity compared with the control group (*p*<0.05). AH Plus® and MTA Fillapex® did not present significant differences between one another; nor were differences found between BioRoot™ RCS and Bio-C® Sealer.

## Discussion

According to the results obtained in the present study, the hypothesis that there are no significant differences in cytotoxicity between different sealers was rejected. The cytotoxicity of the sealers investigated varied from severe to none at all; Bio-C® Sealer showed moderate (tending to slight) cytotoxicity compared with the control group.

Cytotoxicity was assessed by evaluating the effects of the sealers on NIH 3T3 fibroblasts (2,3,14), selected because of the minimum number of steps required to cultivate them and the few alterations they undergo during manipulation. Fibroblasts are the major constituents of connective tissue and the predominant cell type of the periodontal ligament that will be in contact with endodontic sealers. MTS reactive was used to evaluate cell viability as an alternative to the MTT test. The latter produces a formazan precipitate that must be dissolved in DMSO, isopropanol, acid or SDS before measuring its absorbance (1,3,14-17). In this way, the MTS assay used in the present study simplified procedures due to the fact that the formazan produced is soluble in water and does not need additional solvents. This eliminates a liquid handling step during the assay procedure and so saves time and avoids potential error such as the cell loss that can occur when removing culture medium and subsequently solubilizing cells.

Regarding cell morphology, cells that suffer apoptosis exhibit cytoplasmic contraction, nuclear condensation, internucleosomal DNA excision and cell fragmentation (18). Some authors use cell morphology as the sole criterion to identify cell death, but others consider that this criterion is insufficient to affirm that a cell has undergone apoptosis or not. So, in addition to examining cell morphology, they also use a variety of quantitative biochemical assays to evaluate apoptosis directly such as DNA content, caspase 3 activity, and FAK excision (18). In the present work, morphological analysis was supported by the cytotoxicity evaluations obtained.

AH Plus® is the mostly widely investigated sealer in cytotoxicity studies and is often regarded as a reference sealer. In the present work it was found to present the highest cytotoxicity among the evaluated sealers, this is observed in other studies (19,20). Many articles in the literature have observed cytotoxicity when sealer has been recently mixed but that this disappears when the sealer has set and over time (3,21-24). However, one study found that AH Plus® exhibited no cytotoxicity after 24 hours but that cytotoxicity increased to a moderate level within 48 hours and to severe after 72 hours (1), while others found no cytotoxicity compared with a control group (14). The toxicity of AH Plus® is related to formaldehyde release by the amines present in its composition, which aim to accelerate epoxy resin setting, and to components such as bisphenol A, known for its toxicity (24,25). The disparities between studies may be attributed to methodological differences, such as the materials’ setting conditions (whether materials were recently mixed or totally set), the sealers’ concentration (whether it was in a solution or not), exposure time (26), the type of cells and cytotoxicity test used. In the present study, the sealers were totally set, underwent no dissolution and the exposure time was 24 hours.

MTA Fillapex® showed severe cytotoxicity in comparison with controls, a finding that agrees with several studies (1,3,14,21,24,27), but disagrees with the cited studies when compared with AH Plus®. In these studies, MTA Fillapex® was found to be more cytotoxic than AH Plus®, while in the present work the opposite was observed although the difference in cytotoxicity between the two sealers was not statistically significant. One study showed moderate to low cytotoxicity for MTA Fillapex® but this depended on its concentration (15). The cytotoxicity of MTA Fillapex® is related to the presence of salicylate resin, diluting resin, and silica in its composition, and probably to an unbalanced relation between resins and MTA with higher proportions of salicylate resin (14).

BioRoot™ RCS showed moderate-slight cytotoxicity, a finding that partially agrees with several studies, which have reported an absence of cytotoxicity (28,29) or absence of cytotoxicity during the first 24 hours changing to slight cytotoxicity at 48-72 hours (1). Other authors concur with the present findings obtaining slight cytotoxicity values (2,22) and good results in terms of biocompatibility and bioactivity (4).

GuttaFlow® Bioseal showed the best results in terms of cell viability with no statistically significant differences in comparison with the control group. This coincides with previous published articles, which have found high cell viability with this sealer (8) and greater biocompatibility than AH Plus® and MTA Fillapex®.

Regarding Bio-C® Sealer, the obtained results are similar to BioRoot™ RCS, showing moderate-slight cytotoxicity compared to the control group.

Actual studies also suggest that Bio-C® Sealer has better cytocompatibility in comparison with AH Plus® (9,16) and MTA Fillapex (30). However, there are no comparative cytotoxicity studies with the other sealers studied in the present paper.

The cytotoxicity results of AH Plus®, MTA Fillapex® and BioRoot™ RCS are consistent with the literature (1,3,14,19-22,24,27). GuttaFlow® Bioseal presented the best biocompatibility, non-cytotoxic, meaning that no statistically significant differences with the control group are found. Sealers based solely on calcium silicate tend to be the most biocompatible, however GuttaFlow® Bioseal, which is silicone-based combined with calcium silicate particles, presents the lowest cytotoxicity. Bio-C® Sealer showed better biocompatibility than AH Plus® (9,16) and MTA Fillapex® (30) but it cannot be compared with other cytotoxicity studies of the rest of sealers studied in the present paper. Further investigations of these calcium silicate-based sealer are needed to determine its effects in terms of cytotoxicity, both *in vitro* and *in vivo*.

## Conclusions

In the present study Bio-C® Sealer showed moderate (tending to slight) cytotoxicity compared with the control group, GuttaFlow® Bioseal showed no cytotoxicity, BioRoot™ RCS moderate-slight cytotoxicity and AH Plus® and MTA Fillapex® severe cytotoxicity.
